# Efficient generation of gene-modified pigs via injection of zygote with Cas9/sgRNA

**DOI:** 10.1038/srep08256

**Published:** 2015-02-05

**Authors:** Yong Wang, Yinan Du, Bin Shen, Xiaoyang Zhou, Jian Li, Yu Liu, Jianying Wang, Jiankui Zhou, Bian Hu, Nannan Kang, Jimin Gao, Liqing Yu, Xingxu Huang, Hong Wei

**Affiliations:** 1 Department of Laboratory Animal Science, College of Basic Medical Sciences, Third Military Medical University, Chongqing 400038, China; 2 MOE Key Laboratory of Model Animal for Disease Study, Model Animal Research Center of Nanjing University, National Resource Center for Mutant Mice, Nanjing 210061, China; 3 State Key Laboratory of Reproductive Medicine, Department of Histology and Embryology, Nanjing Medical University, Nanjing 210029, China; 4 Department of Immunology, College of Basic Medical Sciences, Third Military Medical University, Chongqing 400038, China; 5 School of Laboratory Medicine, Wenzhou Medical University, Wenzhou, Zhejiang 325035, China; 6 Department of Animal and Avian Sciences, University of Maryland, College Park, MD 27042, USA; 7 Shanghai Key Laboratory of Reproductive Medicine, Shanghai 200025, China

## Abstract

Co-injection of zygotes with Cas9 mRNA and sgRNA has been proven to be an efficient gene-editing strategy for genome modification of different species. Genetic engineering in pigs holds a great promise in biomedical research. By co-injection of one-cell stage embryos with Cas9 mRNA and *Npc1l1* sgRNA, we achieved precise *Npc1l1* targeting in Chinese Bama miniature pigs at the efficiency as high as 100%. Meanwhile, we carefully analyzed the *Npc1l1* sgRNA:Cas9-mediated on- and off-target mutations in various somatic tissues and ovaries, and demonstrated that injection of zygotes with Cas9 mRNA and sgRNA is an efficient and reliable approach for generation of gene-modified pigs.

Pigs are considered as one of the best animals for generating models of human diseases and for providing organs for xenotransplantation, because they share similar features with humans in physiology, anatomy, and lifespan. Genetically modified pigs have been demonstrated to hold a great promise in biomedical research^1^,^2^. However, precise gene modifications of the pig genome are challenging, because traditional gene modifications by homologous recombination (HR) have only been demonstrated to be efficient in authentic embryonic stem cells (ESC) that are not available in pigs^3^. Historically, specific gene knockout strategies in pigs were based on DNA homologous recombination (HR) in somatic cells, followed by somatic cell nuclear transfer (SCNT)^4^.

However, the efficiency of HR in somatic cells is extremely low, which is one of the main barriers in gene targeting in pigs^5^,^6^. Recently, programmable endonucleases, such as Zinc Finger Nucleases (ZFNs), Transcription activator-like effector nucleases (TALENs), and especially the CRISPR, the recently developed clustered regularly interspaced short palindromic repeats (CRISPR)/CRISPR-associated (Cas) 9 system, were shown to be a kind of revolutionary technologies for targeted genome editing^7^. Indeed, these technologies greatly advanced the gene targeting in pigs by HR-based SCNT^8^,^9^,^10^,^11^,^12^,^13^,^14^,^15^. Nevertheless, SCNT is limited by the technical challenges, such as that the porcine fetal fibroblasts used as donors for SCNT have a finite proliferative capacity^16^, SCNT sometimes results in abnormal animals, and the efficiencies of producing offspring by SCNT are low^5^,^17^. Hence, more efficient and reliable strategies are required for gene targeting in pigs. Genome modifications by direct injection of zygotes with DNA or mRNA of ZFNs and TALENs opened a new avenue for precise gene modification in different species^18^,^19^,^20^. This strategy has been successfully used in pigs^21^.

Compared to ZFNs and TALENs, CRISPR/Cas9-mediated genome engineering is easy to handle, highly specific, efficient, and multiplexable^22^. Taking the advantages of CRISPR/Cas9, we achieved efficient gene targeting in mice, rats, and monkeys by co-injection of one-cell stage embryos with Cas9 mRNA and sgRNAs^23^,^24^,^25^, which encouraged us to extend the application of this strategy to gene targeting in pigs, and especially, to further characterize this strategy by thorough analysis of the Cas9/sgRNA-mediated target modification in different tissues, especially the gonads, although using CRISPR/Cas9 to produce gene-modified pigs via direct injection of zygotes has been proved^26^,^37^.

To do this, the Chinese Bama miniature pig was chosen as the model animals. Chinese Bama miniature pig is one of the widely used large model animals for bio-medical research in China, especially for pharmacological experiments^27^,^28^,^29^, because of its genetic stability, high degree of inbreeding, small size, easiness to handle, and it shares anatomical, metabolic and physiological similarities with humans. *Npc1l1* (Niemann-Pick C1-Like 1) was chosen as the target gene. The results showed, through co-injection of early stage embryos of Chinese Bama miniature pigs with *Npc1l1* sgRNA and Cas9 mRNA, target bi-allelic modifications of pig *Npc1l1* were achieved at the efficiency as high as 100%. Meanwhile, we first characterized the Cas9/sgRNA-mediated on- and off-target mutation in various somatic tissues and ovaries, and first provided detailed information about injection of zygotes with Cas9 mRNA and sgRNA. We demonstrated that this CRISPR/Cas9-based approach is efficient and reliable for generation of gene-modified pigs.

## Results and Discussion

### The Design and Validation of sgRNA Targeting Pigs

**Figure 1:**
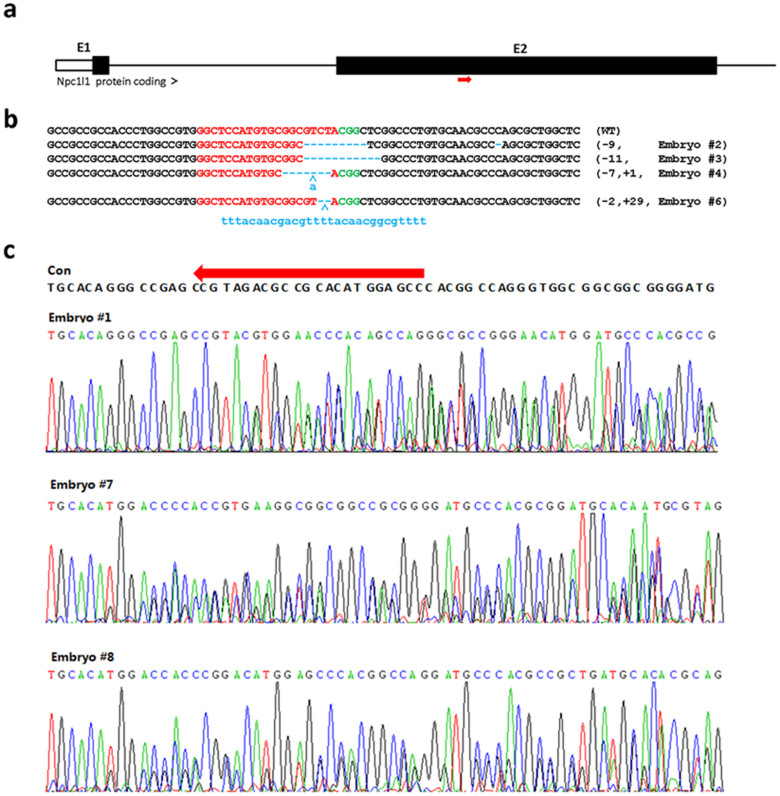
Evaluation of *Npc1l1* sgRNA:Cas9-mediated modifications of *Npc1l1* in pig parthenogenetic embryos. (a) Schematic diagram of pig *Npc1l1* partial protein coding region and the targeting locus of *Npc1l1* sgRNA:Cas9. Red arrow indicates the targeting site of *Npc1l1* sgRNA:Cas9. (b) Sequencing results of the modified *Npc1l1* alleles detected in injected parthenogenetic embryos. Sequences complementary to sgRNA are labeled in red, and PAM sequences are in green; mutations, blue, lower case; deletions, (−); insertions, (+). (c) The chromatographs of sequencing modified *Npc1/1* alleles in parthenogenetic embryos in which overlapped peaks were observed. Con denotes wild-type sequence. Red arrow indicates the targeting site of *Npc1l1* sgRNA:Cas9.

NPC1L1 is highly expressed in small intestine and plays a critical role in both dietary cholesterol absorption and biliary cholesterol reabsorption^30^,^31^,^32^. In mice, NPC1L1 is exclusively expressed in small intestine and gallbladder, but human NPC1L1 is highly expressed in liver, intestine and perhaps other tissues. The tissue distribution of NPC1L1 expression in pigs was unknown. Considering similarities in physiology and metabolism between pigs and humans, we generated NPC1L1-deficient pig models for future definition of how NPC1L1 influences cardiovascular and metabolic diseases. One sgRNA targeting the exon 2 of *Npc1l1* (Figure 1a) was designed as described previously^22^.

Although Cas9 mRNA and sgRNA have been successfully applied to embryos of several species^33^, they had not been tested in pigs when this work was initiated. But the fact that microinjection of zygotes with ZFN or TALEN generated knockout pigs^21^, suggested it was highly possible that Cas9 mRNA and sgRNA would work in pig embryos. To determine if Cas9 mRNA and sgRNA system works in pig embryos, we transcribed the Cas9 and *Npc1l1* sgRNA *in vitro* using T7 RNA polymerase as described previously^23^. Twenty nanograms of Cas9 mRNA and 10 nanograms of sgRNA were pooled and microinjected into cytoplasm of 195 pig parthenogenetic embryos as described by *Wang et al.* 39. (Table 1). A total of 28 embryos normally developed to blastocyst at even a slightly higher efficiency of cleavage (the experimental group: 52.8% (103/195) v.s. the control group: 42.4% (91/225); P = 0.014). The blastocyst development rate in the experimental group was comparable to that of the control group (14.4% (28/195) v.s. 13.3% (30/225); P = 0.778; Table 1), suggesting that Cas9 mRNA and *Npc1l1* sgRNA were not toxic to the embryos of pigs.

We isolated the pig genomic DNA samples from a total of 8 individual embryos at 144 hr post microinjection, and screened for the presence of site-specific gene modification by PCR amplification of the region around the target site followed by T7EN1 cleavage assays using the PCR products as substrates. The cleavage bands were visible in the target gene (Data not shown). Further characterization of the cleavage by sequencing the PCR products revealed that 3 out of 8 samples displayed overlapped peaks in the sequencing chromatographs (test embryos #1, #7 & #8) and 4 out of 8 samples exhibited distinguishable insertions or deletions (indels) with variable mutation sizes (−11 ~ +29 bp) (test embryos #2, #3, #4 & #6) at the target site (Figure 1b & c, and Table 2). These data demonstrated that the selected sgRNA worked well with Cas9 on the target gene in pig embryos.

### Cas9/sgRNA Enables One-Step Gene Modification in Pigs

**Figure 2:**
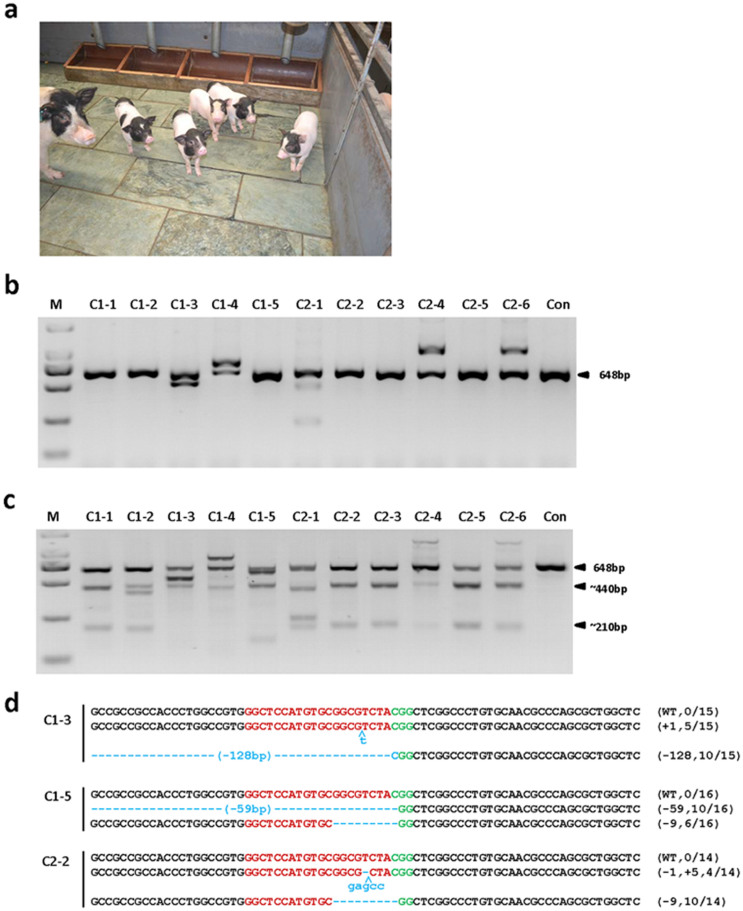
Detection of *Npc1l1*sgRNA:Cas9-mediated modifications of *NPC1L1* in founder pigs. (a) A photo showing 38-day-old pigs carrying *Npc1l1* mutations. (b) PCR products of the targeted region of *Npc1l1* from founder pigs. The first litter of five live pigs were named C1-1 to C1-5. The second litter of six live pigs were named from C2-1 to C2-6. Con denotes wild-type pig as a control. Notably, the PCR products of C1-3, C1-4, C2-1, C2-4 and C2-6 differ from the control in size, suggesting a large fragment deletion or insertion. (c) Detection of *Npc1l1* sgRNA:Cas9-mediated on-target cleavage of *Npc1l1* by T7EN1 cleavage assays. All PCR products from (b) were subjected to T7EN1 cleavage assays. All the samples were digested by T7EN1, suggesting that all founders carry *Npc1l1* mutations. (d) Sequencing results of modified *Npc1l1* alleles detected in founder pigs. At least 12 TA clones of the PCR products were analyzed. Sequences complementary to sgRNA are labeled in red, and PAM sequences in green. Mutations, blue, lower case; deletions, (−); insertions, (+). N/N indicates positive colonies out of total sequenced samples. See also Figure S1.

With the success in pig parthenogenetic embryos, we set out to generate knockout Chinese Bama miniature pigs. A total of 110 porcine early embryos (1-cell stage) were surgically collected from 20 mated sows. The Cas9 mRNA and *Npc1l1* sgRNA mixtures were injected into cytoplasm of embryo cells as described above. A total of 105 out of 110 injected embryos were transferred into 4 surrogate females. Of the 4 recipient mothers, 2 pregnancies were established (50%; 2 out of 4). After full-term (~114 days) pregnancy, 2 litters (Litter 1: C1-1 ~ C1-5; Litter 2: C2-1 ~ C2-6) of 12 piglets were successfully delivered alive, but one died immediately after birth (Figure 2a and Table 3).

To genotype the piglets, the ear punch tissues of the eleven live infant pigs were collected and the genomic DNA was isolated. The *Npc1l1* sgRNA:Cas9-mediated genome modifications were first screened by PCR and T7EN1 cleavage assay using the genomic DNA as described above. The additional bands were observed by PCR amplification of the target region in 5 infants, including C1-3, C1-4, C2-1, C2-4, and C2-6 (Figure 2b), suggesting that the genomic modification occurred in these founder animals with large indels. Then, the PCR products of all the founders were subjected to the T7EN1 cleavage assay (Figure 2c). Very excitingly, the cleavage products were observed in all the 11 infants, indicating efficient genomic modifications in the founder piglets. As expected, different kinds of indels were detected by sequencing the PCR products (Figure 2d, Table 4, and Supplementary Figure 1), further confirming the efficient genomic modifications. Incredibly, no wild-type sequence was detected in all of the founders, indicating that CRISPR/Cas9 induced bi-allelic mutations at an extremely high efficiency in pig embryos.

### Cas9/sgRNA-Mediated Genome Targeting Extensively Integrates into Different Tissues of Pigs

**Figure 3:**
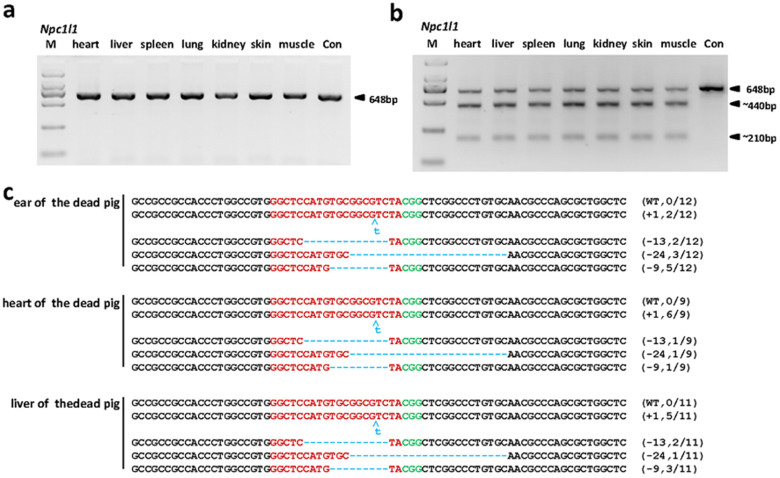
Detection of *Npc1l1* sgRNA:Cas9-mediated targeting in different tissues. (a) PCR products of the targeted region of *Npc1l1* from 7 different tissues. (b) Detection of *Npc1l1* sgRNA:Cas9-mediated on-target cleavage of *Npc1l1*. All PCR products from (a) were subjected to T7EN1 cleavage assays. (c) Sequencing results of modified *Npc1l1* alleles detected in ear, heart, and liver. Sequences complementary to sgRNA are labeled in red, and PAM sequences in green. Mutations, blue, lower case; deletions, (−); insertions, (+). N/N indicates positive colonies out of total sequenced.

All of the targeting results described above were from the noninvasively available ear tissues. The dead founder pig provided us an opportunity to evaluate the integration of the Cas9/sgRNA-mediated *Npc1l1* targeting into the derivatives of three germ layers, which generated detailed information for CRISPR/Cas9-mediated genome targeting in pigs via injection of zygotes with Cas9/sgRNA mixture. By PCR amplification and T7EN1 cleavage assays, we first performed extensive analysis of the target mutagenesis in 7 different somatic tissues, including heart, liver, spleen, lung, kidney, skin, and muscle from the dead founder. While the PCR bands did not differ between experimental and control groups (Figure 3a), but the T7EN1 assays showed that the cleavage bands existed in every reactions (Figure 3b), indicating that the Cas9/sgRNA-mediated mutations occurred in all tissues examined. The target mutations were further confirmed by sequencing the samples from ear, heart, and liver, which are derived from ectoderm, mesoderm, and endoderm, respectively, and the results showed that identical genetic modifications extensively integrated into all three germ layers (Figure 3c and Table 5). Again, no wild type allele was detected, indicating that CRISPR/Cas9-mediated bi-allelic mutations existed in all tissues examined. These results further substantiate the high efficiency of CRISPR/Cas9 system in pigs.

### Cas9/sgRNA-Mediated Genome Targeting Efficiently Transmits into Ovaries of Pigs

**Figure 4:**
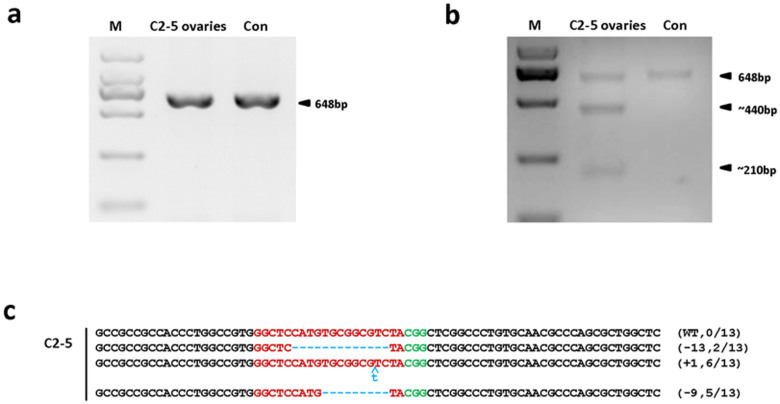
*Npc1l1* sgRNA:Cas9-mediated modifications in the ovaries of founder C2-5. (a) PCR products of the targeted region of *Npc1l1* in the ovaries of founder C2-5. (b) Detection of *Npc1l1* sgRNA:Cas9-mediated cleavage of *Npc1l1* in ovaries. All PCR products from (a) were subjected to T7EN1 cleavage assays. (c) Sequencing results of modified *Npc1l1* alleles detected in the ovaries of founder C2-5. PCR products of the targeted region of *Npc1l1* were amplified from the ovaries and sequenced. The PAM sequences are highlighted in green; the targeting sequences in red; the mutations are shown in blue, lower case. Deletions, (−); and Insertions, (+). N/N indicates positive colonies out of total sequenced.

To establish genetically modified animals requires germline transmission of genetic mutations. Because the gene-modified Bama miniature pigs have not reached sexual maturity, we currently cannot perform breeding to determine germline transmission. But the highly efficient occurrences of the target mutations in different tissues strongly suggest it is very possible that the Cas9/sgRNA-mediated genome targeting will integrate into the pig gonads. To prove the possibility, the founder C2-5 was sacrificed and the ovaries were isolated. The examination of the target mutagenesis was performed as above. The PCR and T7EN1 assays revealed that the cleavage of *Npc1l1* did occur in the ovaries (Figure 4a & b). This was confirmed by sequencing of the target mutations, which were identical to those detected in the somatic tissues, such as a 1-bp insertion, 9-, 13-, and 24-bp deletions (Figure 4c and Table 6), and absence of wild-type sequence, demonstrating the Cas9/sgRNA-mediated genome targeting successfully integrated into the gonads too. It is notifying, the gonads are composed of germ cells and somatic cells. The sequencing results of the ovaries showed that the efficiency of Cas9/sgRNA-induced targeting was 100% in the ovaries, strongly suggesting that the Cas9/sgRNA-mediated targeting was most likely transmitted into the pig germ cells. Nevertheless, breeding results will provide direct evidence of germline transmission in the future.

### Mosaicism Analysis

The sequence data of both live and dead founder animals revealed multiple genotypes (Figure 2d, 3c, and 4c; Table 4 ~ 6 and Supplementary Figure 1), suggesting that the CRISPR/Cas9-mediated cleavage had occurred multiple times and resulted in mosaicism of the modification as seen in other species^23^,^24^,^25^.

Further characterization of the mosaicism showed that a total of 18 kinds of indels with mutation sizes ranging from −412 bp to +535 bp were detected in different founders. Surprisingly, the 9 bp deletions were detected in 8 of the 11 founders, suggesting the possible preference of the Cas9/sgRNA-mediated *Npc1l1* targeting. Of the total 11 founder piglets, 6 (C1-1, C1-2, C1-3, C1-5, C2-2, and C2-4) harbored with 2, 3 (C1-4, 2-1 and C2-3) with 3, and 2 (C2-5 and C2-6) with 4 different modifications (Table 4). Furthermore, the same modifications were detected in different tissues (Figure 3 and Table 4 & 5, Supplementary Figure 1) and ovaries (Figure 4c; Table 6 and Supplementary Figure 1) of the same founder, indicating that the modification occurred at very early embryogenesis.

### Off-Target Detection

**Figure 5:**
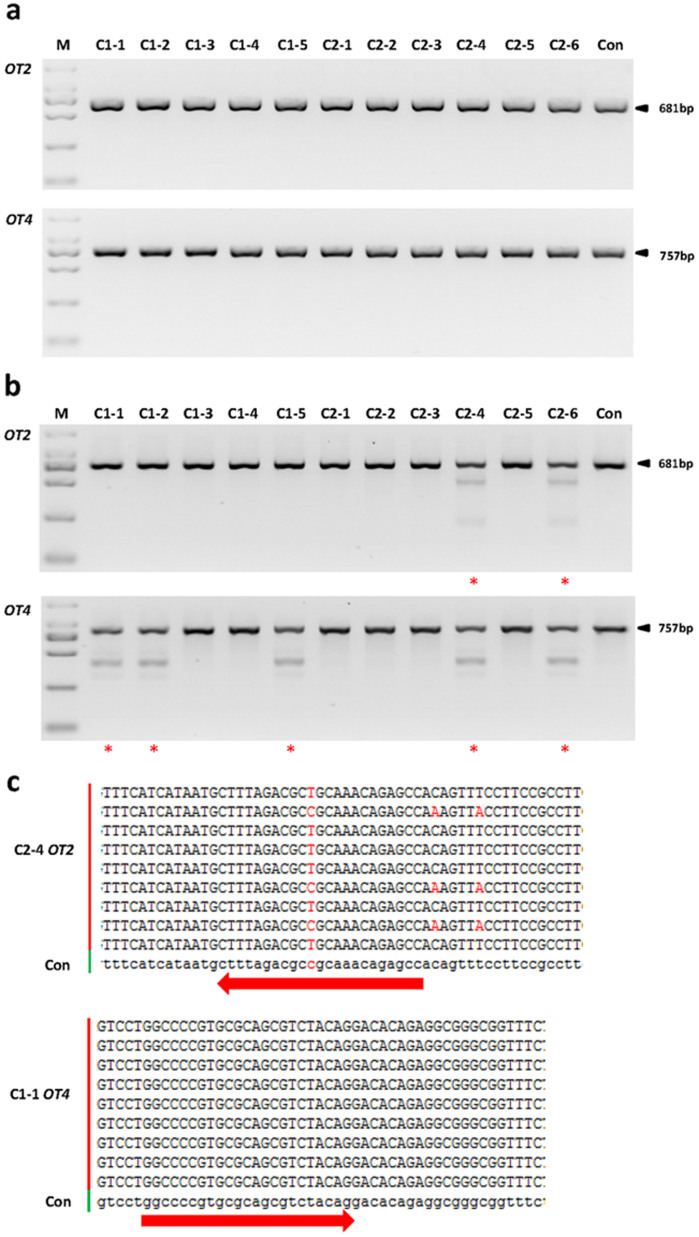
Detection of the *Npc1l1* sgRNA:Cas9-mediated off-target cleavages *in vivo*. (a) PCR products of the potential off-target sites 2 and 4 (OTs 2 and 4) of *Npc1l1* sgRNA:Cas9 from founder pigs. (b) Detection of *Npc1l1* sgRNA:Cas9-mediated off-target cleavage at the OTs 2 and 4. All PCR products from (a) were subjected to T7EN1 cleavage assays. *, different cleavage band patterns compared to the control. The size of the digested bands does not match that expected. (c) Sequencing results of PCR products. No off-target indel mutation was observed in the T7EN7 cleavage bands. The two red arrows indicate potential off-target sites of OT2 and OT4, respectively. Con denotes the corresponding sequence of each retrieved from Ensembl.

Off-target mutagenesis is of a major concern for CRISPR/Cas9 system^33^,^34^,^35^. The CRISPR/Cas9-induced off-target mutation is heritable both in mice and rats^23^,^24^. We thus ask whether off-target mutation occurred in our genetic modified pigs. To this end, we screened the pig genome using the open SeqMap tool, and predicted a total of 8 most potential off-target sites (OT 1 ~ 8) (Supplementary Table 1). Using the primers listed in Supplementary Table 4, all the selected OTs were subjected to PCR amplification and T7EN1 cleavage assay. Results showed that except the OTs 2 and 4, no genetic modification was detected at the selected OTs (Figure 5a & b, Supplementary Figure 2). At the OTs 2 and 4, distinguishable cleavage bands of unexpected size were detected in 7 reactions of founders C1-1, C1-2, C1-5, C2-4, C2-6, including 2 (Founders C2-4 and C2-6) for OT2, and 5 (Founders C1-1, C1-2, C1-5, C2-4 and C2-6) for OT 4 (Figure 5b), indicating that the *Npc1l1* sgRNA:Cas9 might have induced non-specific mutations at these two sites.

To confirm the *Npc1l1* sgRNA:Cas9-mediated off-target cleavage events, PCR products from C1-1 for OT2 and C2-4 for OT 4 were randomly chosen for sequencing. Surprisingly, no real off-target mutations were detected, and the unexpected cleavages came from SNPs (Figure 5c). These results demonstrated that CRISPR/Cas9 did not induce detectable off-target mutations in this study. Nevertheless, more comprehensive study may be required to clarify the off-target effects of these pigs. Considering the off-target mutagenesis is site-dependent, and more specific strategies for organisms using mutated Cas9 has already been established^36^, the off-target mutagenesis can be minimized by optimizing the procedure.

In summary we show here that the site-specific pig gene modification can be efficiently achieved by co-injection of Cas9 mRNA and sgRNAs into the one-cell embryos. Furthermore, by careful characterization of the *Npc1l1* sgRNA:Cas9-mediated on- and off-target effects, the mosacism, *et al*., we first provide detailed information about the Cas9/sgRNA-mediated target mutations via direct injection of zygotes in various somatic tissues, as well as the gonads, confirming the CRISPR/Cas9 system is versatile for heritable pig genome targeting without detectable off-target mutagenesis. During preparation of this manuscript, a similar study was reported^37^, which supports our conclusion that direct injection of zygotes with Cas9/RNA is an efficient and reliable approach for pig genome targeting.

## Methods

### Animals

The animals used in this study were regularly maintained in the Laboratory Animal Centre of the Third Military Medical University. All the protocols involving the use of animals were in accordance with approved guidelines of the Institutional Animal Care and Use Committee of the Third Military Medical University (Approval ID: SYXK-PLA-2007036).

### Vector constructs and *in vitro* transcription

To construct the recombinant vector for preparation of sgRNA by *in vitro* transcription, the two complementary DNA oligos shown in Supplementary Table 2 were annealed to be double-stranded and subcloned into pUC57-T7-gRNA vector as described^23^. Using the constructed recombinant vector that was completely linearized by the endonuclease DraI as the templates, sgRNAs were produced via *in vitro* transcription using MEGAshortscript kit (Ambion) and purified using MEGAClear kit (Ambion) as described in the manuals. Using the Cas9 mRNA *in vitro* transcription vector (Addgene No. 44758) as templates, Cas9 mRNAs were produced and purified as described previously by Shen *et al.*^23^.

### Cas9/sgRNA efficacy test via pig parthenogenetic embryo injection

Pig cumulus-oocyte complexes (COCs) were harvested from ovaries collected from slaughter houses. The COCs were cultured for *in vitro* maturation as described^5^,^38^,^39^. The pig oocytes were freed of cumulus by treating the cultured COCs with hyaluronidase, and the matured oocytes with extruded polar body were selected out and subjected to cytoplasmic microinjection with Cas9/sgRNA mixture as described^39^. The injected oocytes were activated by direct current electrical pulses (1.2 KV/cm, 30 μs, two times, 1 sec interval) and the activated oocytes (parthenogenetic embryos) were cultured in PZM-3 media as described by Wang *et al.*^39^. The cleavage rate of parthenogenetic embryos was counted at 48 hr post activation and blastocystes harvested at 144 hr post activation. Pig genomic DNA was extracted from single parthenogenetic blastocysts by incubating individual embryos in lysis buffer as described^39^. Using the genomic DNAs as templates, a primer pair set (p*Npc1l1*-exon2-sgR-F: 5-GACCTACGAGTCCTGCAGC-3; p*Npc1l1*-exon2-sgR-R: 5-GAAGACCGAGCAGAGGATGA-3; product size: 386 bp) were used to amplify modified *Npc1l1* alleles in injected embryos by PCR, and the amplification products were subjected to T7EN1 analysis or sequencing after purification using gel extraction kit (Qiagen).

### Production of gene-modified pigs via zygote injection with Cas9/sgRNA

Cas9 mRNA and sgRNA were mixed at the final concentrations of 20 and 10 ng/μL, respectively. Pig zygotes were surgically collected from mated sows as described^40^. The collected zygotes were subjected to cytoplasmic microinjection with the Cas9/sgRNA mixture in the same way as that for parthenogenetic embryos described above. Shortly after injection, the injected zygotes were transferred into synchronized foster mother sows as described^40^. Pregnancy was investigated by observing the oestrus behaviors of recipient sows at every ovation circle.

### T7EN1 cleavage assay and sequencing

Different samples were collected and digested in a lysis buffer (0.4 M NaCl, 2 μM EDTA, 1% SDS, 10 μM Tris-HCl, and 100 μg/ml Proteinase K). The genomic DNA of the sample was extracted from lysate by phenol-chloroform, and recovered by alcohol precipitation. T7EN1 cleavage assay was performed as described by Shen *et al*^23^. Briefly, the targeted fragments were amplified by PrimerSTAR HS DNA polymerase (TaKaRa, DR010A) from the genomic DNA, then purified with a PCR cleanup kit (Axygen, AP-PCR-50). The primers for amplifying *Npc1l1* targeted fragments were listed in Supplementary Table 3. The purified PCR product was denatured and re-annealed in NEBuffer 2 (NEB) using a thermocycler. The PCR products were digested with T7EN1 (NEB, M0302L) for 30 min at 37°C and separated on a 2.5% agarose gel. The PCR products with mutations detected by T7EN1 cleavage assay were sub-cloned into T vector (Takara, D103A). For each sample, the colonies were picked up randomly and sequenced by M13F (−47) primer (M13F (−47): 5′-CGC CAG GGT TTT CCC AGT CAC GAC-3′).

### Off-target assay

To determine the site-specific cleavage of the CRISPR-Cas9 system *in vivo*, the potential off-target loci were searched by an open tool, SeqMap^41^. The mismatch parameter of target sequence was set as described^25^. ‘NGG’ and ‘NAG’ were chosen as PAM. The sites that have conserved 7 bp proximal to PAM with total mismatches < 5 and the sites with total mismatches < 4 were chosen as potential off-target sites for subsequent test. The selected potential off-target sites were PCR amplified using genomic DNA as templates. The PCR products were first subjected to T7EN1 cleavage assay. The potential off-target sites yielding typical cleavage bands were considered as candidates. The PCR products of the candidates were cloned and sequenced to confirm the off-target effects. The primer pairs used were listed in Supplementary Table 4.

### Statistical analysis

Chi-square Test was performed in two-tailed manner to evaluate the difference of parthenogenetic embryo cleavage rate and blastocyst development rate between the Cas9/sgRNA injection and untreated groups.

## Author Contributions

H.W., X.H., J.G. and L.Y. designed the experiments and wrote the manuscript. Y.W., Y.D., B.S., X.Z., J.L., Y.L., J.W., J.Z., B.H. and N.K. performed the experiments.

## Supplementary Material

Supplementary Information Wang et al., Supplementary information

## Figures and Tables

**Table 1:** Summary of test embryo microinjection of Cas9 mRNA and *Npc1l1*sgRNA

Number of collected ovaries	Number of cultured COCs	Number of mature oocytes	Number of Cas9/sgRNA injection oocytes		Cleavage rate at 48 hr post activation		Blastocyst development rate at 144 hr post activation	
			Injected group	Untreated group	Injected group	Untreated group	Injected group	Untreated group
400	810	420	195	225	52.8% (103/195)^a^	40.4% (91/225)^b^	14.4% (28/195)^a^	13.3% (30/225)^a^

Note: The a and b indicate statistical significance.

**Table 2:** Summary of *Npc1l1* alleles from test embryos with *Npc1l1* sgRNA:Cas9-mediated modifications

Embryo No.	Target site mutations
#1	overlapped peaks
#2	del 9 bp
#3	del 11 bp
#4	del 7 bp, insert 1 bp
#5	WT
#6	del 2 bp, insert 29 bp
#7	overlapped peaks
#8	overlapped peaks

Del: deletion; insert: insertion.

**Table 3:** Summary of embryo microinjection of Cas9 mRNA and *Npc1l1* sgRNA

	Injected mixture	Injected embryos	Transferred embryos	Recipient amount	Established pregnancy	Piglets born
1st	10 ng *Npc1l1-*sgRNA + 20 ng Cas9 mRNA	27	27	1	1	5 (live,C1-1~5)+1 (dead)
2nd	10 ng *Npc1l1*-sgRNA + 20 ng Cas9 mRNA	36	32	1	1	6 (live, C2-1~6)
3rd	10 ng *Npc1l1*-sgRNA + 20 ng Cas9 mRNA	23	23	1	0	0
4th	10 ng *Npc1l1*-sgRNA + 20 ng Cas9 mRNA	24	23	1	0	0
Total		110	105	4	2	12

**Table 4:** Summary of alleles from founders with *Npc1l1* sgRNA:Cas9-mediated modifications

Founder No.	Target site mutations	Sequenced colonies
C1-1	del 24 bp	8
	del 9 bp	6
C1-2	del 16 bp	7
	del 9 bp	8
C1-3	insert 1 bp	5
	del 128 bp	10
C1-4	del 9 bp	1
	del 89 bp	10
	del 104 bp, insert 270 bp	2
C1-5	del 59 bp	10
	del 9 bp	6
C2-1	del 42 bp, insert 56 bp	5
	del 24 bp	5
	del 412 bp	4
C2-2	del 1 bp, insert 5 bp	4
	del 9 bp	10
C2-3	del 24 bp, insert 7 bp	3
	del 12 bp	2
	del 9 bp	9
C2-4	del 9 bp	12
	del 6 bp, insert 535 bp	1
C2-5	del 13 bp	6
	insert 1 bp	2
	del 9 bp	3
	del 24 bp	1
C2-6	del 28 bp	3
	del 24 bp	2
	del 9 bp, insert 2 bp	7
	del 12 bp, insert 509 bp	1

Del: deletion; insert: insertion.

**Table 5:** Summary of Npc1l1 alleles from different tissues with *Npc1l1* sgRNA:Cas9-mediated modifications

Tissues	Target site mutations	Sequenced colonies
ear	insert 1 bp	2
	del 13 bp	2
	del 24 bp	3
	del 9 bp	5
heart	insert 1 bp	6
	del 13 bp	1
	del 24 bp	1
	del 9 bp	1
liver	insert 1 bp	5
	del 13 bp	2
	del 24 bp	1
	del 9 bp	3

Del: deletion; insert: insertion.

**Table 6:** Summary of alleles from ovaries of C2-5 founder with *Npc1l1* sgRNA:Cas9-mediated modifications

	Target site mutations	Sequenced colonies
Ovaries of C2-5	del 13 bp	2
	insert 1 bp	5
	del 9 bp	4
	del 24 bp	2

Del: deletion; insert: insertion.

## References

[b1] Fahrenkrug S. C. *et al.* Precision genetics for complex objectives in animal agriculture. J. Anim. Sci. 88, 2530–2539 (2010). 20228236 10.2527/jas.2010-2847 PMC7109650

[b2] Tan W. S., Carlson D. F., Walton M. W., Fahrenkrug S. C. & Hackett P. B. Precision editing of large animal genomes. Adv. Genet. 80, 37–97 (2012). 23084873 10.1016/B978-0-12-404742-6.00002-8 PMC3683964

[b3] Capecchi M. R. Altering the genome by homologous recombination. Science 244, 1288–1292 (1989). 2660260 10.1126/science.2660260

[b4] Clark A. J. & Whitelaw C. B. A. A future for transgenic livestock. Nat. Rev. Genet. 4, 825–833 (2003). 14526378 10.1038/nrg1183 PMC7097355

[b5] Lai L. *et al.* Production of α-1,3-galactosyltransferase knockout pigs by nuclear transfer cloning. Science 295, 1089–1092 (2002). 11778012 10.1126/science.1068228

[b6] Rogers C. S. *et al.* Disruption of the CFTR gene produces a model of cysticfibrosis in newborn pigs. Science 321, 1837–1841 (2008). 18818360 10.1126/science.1163600 PMC2570747

[b7] Kim H. & Kim J. S. A guide to genome engineering with programmable nucleases. Nat. Rev. Genet. 15, 321–334 (2014). 24690881 10.1038/nrg3686

[b8] Bao L. *et al.* Generation of GGTA1 biallelic knockout pigs via zinc-finger nucleases and somatic cell nuclear transfer. Sci. China Life Sci. 57, 263–268 (2014). 24430555 10.1007/s11427-013-4601-2

[b9] Carlson D. F. *et al.* Efficient TALEN-mediated gene knockout in livestock. Proc. Natl. Acad. Sci. USA 109, 17382–17387 (2012). 23027955 10.1073/pnas.1211446109 PMC3491456

[b10] Hauschild J. *et al.* Efficient generation of a biallelic knockout in pigs using zinc-finger nucleases. Proc.Natl. Acad. Sci. USA 108, 12013–12017 (2011). 21730124 10.1073/pnas.1106422108 PMC3141985

[b11] Kwon D. N. *et al.* Production of biallelic CMP-Neu5Ac hydroxylase knock-out pigs. Sci. Rep. 3, 1981; 10.1038/srep01981 (2013). 23760311 PMC4070623

[b12] Tan W. S. *et al.* Efficient nonmeiotic allele introgressionin livestock using custom endonucleases. Proc. Natl. Acad. Sci. USA 110, 16526–16531 (2013). 24014591 10.1073/pnas.1310478110 PMC3799378

[b13] Xin J. *et al.* Highlyefficient generation of GGTA1 biallelic knockout inbred minipigs with TALENs. PLoS ONE 8, e84250 (2013). 24358349 10.1371/journal.pone.0084250 PMC3866186

[b14] Yang D. *et al.* Generation of PPARγ mono-allelic knockout pigs via zinc-finger nucleases and nuclear transfer cloning. Cell Res. 21, 979–982 (2011). 21502977 10.1038/cr.2011.70 PMC3203707

[b15] Zhou X. *et al.* Generation of CRISPR/Cas9-mediated gene-targeted pigs via somatic cell nuclear transfer. Cell Mol. Life Sci. s00018-014-1744-7; 10.1007/s00018-014-1744-7 (2014). PMC11113635 25274063

[b16] Zhu H. *et al.* Influence of tissue origins and external microenvironment on porcine foetal fibroblast growth, proliferative life span and genome stability. Cell Prolif. 37, 255–266 (2004). 15144502 10.1111/j.1365-2184.2004.00310.x PMC6760691

[b17] Carter D. B. *et al.* Phenotyping of transgenic cloned piglets. Cloning Stem Cells 4, 131–145 (2002). 12171705 10.1089/153623002320253319

[b18] Bogdanove A. J. & Voytas D. F. TAL effectors: customizable proteins for DNA targeting. Science 333, 1843–1846 (2011). 21960622 10.1126/science.1204094

[b19] Carroll D. *et al.* Gene targeting in Drosophila and Caenorhabditis elegans with zinc-finger nucleases. Methods Mol. Biol. 435, 63–77 (2008). 18370068 10.1007/978-1-59745-232-8_5

[b20] Urnov F. D., Rebar E. J., Holmes M. C., Zhang H. S. & Gregory P. D. Genome editing with engineered zinc finger nucleases. Nat. Rev. Genet. 11, 636–646 (2010). 20717154 10.1038/nrg2842

[b21] Lillico S. G. *et al.* Live pigs produced from genome edited zygotes. Sci. Rep. 3, 02847; 10.1038/srep02847 (2013). PMC6505673 24108318

[b22] Mali P., Esvelt K. M. & Church G. M. Cas9 as a versatile tool for engineering biology. Nat. Methods 10, 957–963 (2013). 24076990 10.1038/nmeth.2649 PMC4051438

[b23] Shen B. *et al.* Generation of gene-modified mice via Cas9/RNA-mediated gene targeting. Cell Res. 23, 720–723 (2013). 23545779 10.1038/cr.2013.46 PMC3641603

[b24] Ma Y. *et al.* Generating rats with conditional alleles using CRISPR/Cas9. Cell Res. 24, 122–125 (2014). 24296780 10.1038/cr.2013.157 PMC3879705

[b25] Niu Y. *et al.* Generation of gene-modified cynomolgus monkey via Cas9/RNA-mediated gene targeting in one-cell embryos. Cell 156, 836–843 (2014). 24486104 10.1016/j.cell.2014.01.027

[b26] Whitworth K. M. *et al.* Use of the CRISPR/Cas9 system to produce genetically engineered pigs from in vitro-derived oocytes and embryos. Biol. Reprod. 91, 78 (2014). 25100712 10.1095/biolreprod.114.121723 PMC4435063

[b27] Puccinelli E., Gervasi P. G. & Longo. V. Xenobiotic metabolizing cytochrome P450 in pig, a promising animal model. Current Drug Metabolism 12, 507–525 (2011). 21476973 10.2174/138920011795713698

[b28] Shang H., Yang J., Liu Y. & Wei H. Tissue distribution of CYP3A29 mRNA expressionin Bama miniature pig by quantitative reversetranscriptase-polymerase chain reaction (RT-PCR). Xenobiotica. 39, 423–429 (2009). 19480548 10.1080/00498250902825363

[b29] Liu Y., Zeng B. H., Shang H. T., Cen Y. Y. & Wei H. Bama miniature pigs (Sus scrofa domestica) as a model for drug evaluation for humans: comparison of in vitro metabolism and in vivo pharmacokinetics of lovastatin. Comp. Med. 58, 580–587 (2008). 19149415 PMC2710758

[b30] Altmann S. W. *et al.* Niemann-Pick C1 Like 1 protein is critical for intestinal cholesterol absorption. Science 303, 1201–1204 (2004). 14976318 10.1126/science.1093131

[b31] Temel R. E. *et al.* Hepatic Niemann-Pick C1-like 1 regulates biliary cholesterol concentration and is a target of ezetimibe. J. Clin. Invest. 117, 1968–1978 (2007). 17571164 10.1172/JCI30060 PMC1888567

[b32] Davis H. R. Jr *et al.* Niemann-Pick C1 Like 1 (NPC1L1) is the intestinal phytosterol and cholesterol transporter and a key modulator of whole-body cholesterol homeostasis. J. Biol. Chem. 279, 33586–33592 (2004). 15173162 10.1074/jbc.M405817200

[b33] Sander J. D. & Joung J. K. CRISPR-Cas systems for editing, regulating and targeting genomes. Nat. Biotechnol. 32, 347–355 (2014). 24584096 10.1038/nbt.2842 PMC4022601

[b34] Hsu P. D. *et al.* DNA targeting specificity of RNA-guided Cas9 nucleases. Nat. Biotechnol. 31, 827–32 (2013). 23873081 10.1038/nbt.2647 PMC3969858

[b35] Pattanayak V. *et al.* High-throughput profiling of off-target DNA cleavage reveals RNA-programmed Cas9 nuclease specificity. Nat. Biotechnol. 31, 839–843 (2013). 23934178 10.1038/nbt.2673 PMC3782611

[b36] Shen B. *et al.* Efficient genome modification in mice by CRISPR/Cas9 nickase without off-target effects. Nat. Methods 11, 399–402 (2014). 24584192 10.1038/nmeth.2857

[b37] Hai T., Teng F., Guo R., Li W. & Zhou Q. One-step generation of knockout pigs by zygote injection of CRISPR/Cas system. Cell Res. 24, 372–375 (2014). 24481528 10.1038/cr.2014.11 PMC3945887

[b38] Betthauser J. *et al.* Production of cloned pigs from in vitro system. Nat. Biotechnol. 18, 1055–1059 (2000). 11017042 10.1038/80242

[b39] Wang Y. *et al.* The meganuclease I-sceI containing nuclear localization Signal (NLS-I-sceI) efficiently mediated mammalian germline transgenesis via embryo cytoplasmic microinjection. PLoS ONE 9, e108347 (2014). 25250567 10.1371/journal.pone.0108347 PMC4177210

[b40] Whitelaw C. B. *et al.* Efficient generation of transgenic pigs using equine infectious anaemia virus (EIAV) derived vector. FEBS Letter 571, 233–236 (2004). 10.1016/j.febslet.2004.06.076 15280048

[b41] Jiang H. & Wong W. H. SeqMap: mapping massive amount of oligonucleotides to the genome. Bioinformatics 24, 2395–2396 (2008). 18697769 10.1093/bioinformatics/btn429 PMC2562015

