# Supplementary material for: Efficient generation of gene-modified pigs via injection of zygote with Cas9/sgRNA
Source: Sci Rep. 2015 Feb 5;5:8256. doi: 10.1038/srep08256 (PMC4317696; doi:10.1038/srep08256)
Supplement: Supplementary Information — Wang et al., Supplementary information [file srep08256-s1.pdf]

Efficient generation of gene-modified pigs via injection of zygote with Cas9/sgRNA

Yong Wang[1,8], Yinan Du[2,8], Bin Shen[3],Xiaoyang Zhou[1], Jian Li[4], Yu Liu[1], Jianying Wang[3],Jiankui Zhou[2],Bian Hu[2],Nannan Kang[5], Jimin Gao[5], Liqing Yu[6], Xingxu Huang[2,7*], Hong Wei[1,*]

[1]Department of Laboratory Animal Science, College of Basic Medical Sciences, Third Military Medical University, Chongqing 400038, China.

[2]MOE Key Laboratory of Model Animal for Disease Study, Model Animal Research Center of Nanjing University, National Resource Center for Mutant Mice, Nanjing210061, China

[3]State Key Laboratory of Reproductive Medicine, Department of Histology and Embryology, Nanjing Medical University, Nanjing 210029, China

[4]Department of Immunology, College of Basic Medical Sciences, Third Military Medical University, Chongqing 400038, China.

[5]School of Laboratory Medicine, Wenzhou Medical University, Wenzhou, Zhejiang 325035, China

[6]Department of Animal and Avian Sciences, University of Maryland, College Park, MD 27042, USA

[7]Shanghai Key Laboratory of Reproductive Medicine, Shanghai 200025, China

[8]These authors contributed equally to this work.

*Correspondence: weihong63528@163.com (H.W), xingxuhuang@mail.nju.edu.cn (X.H.)

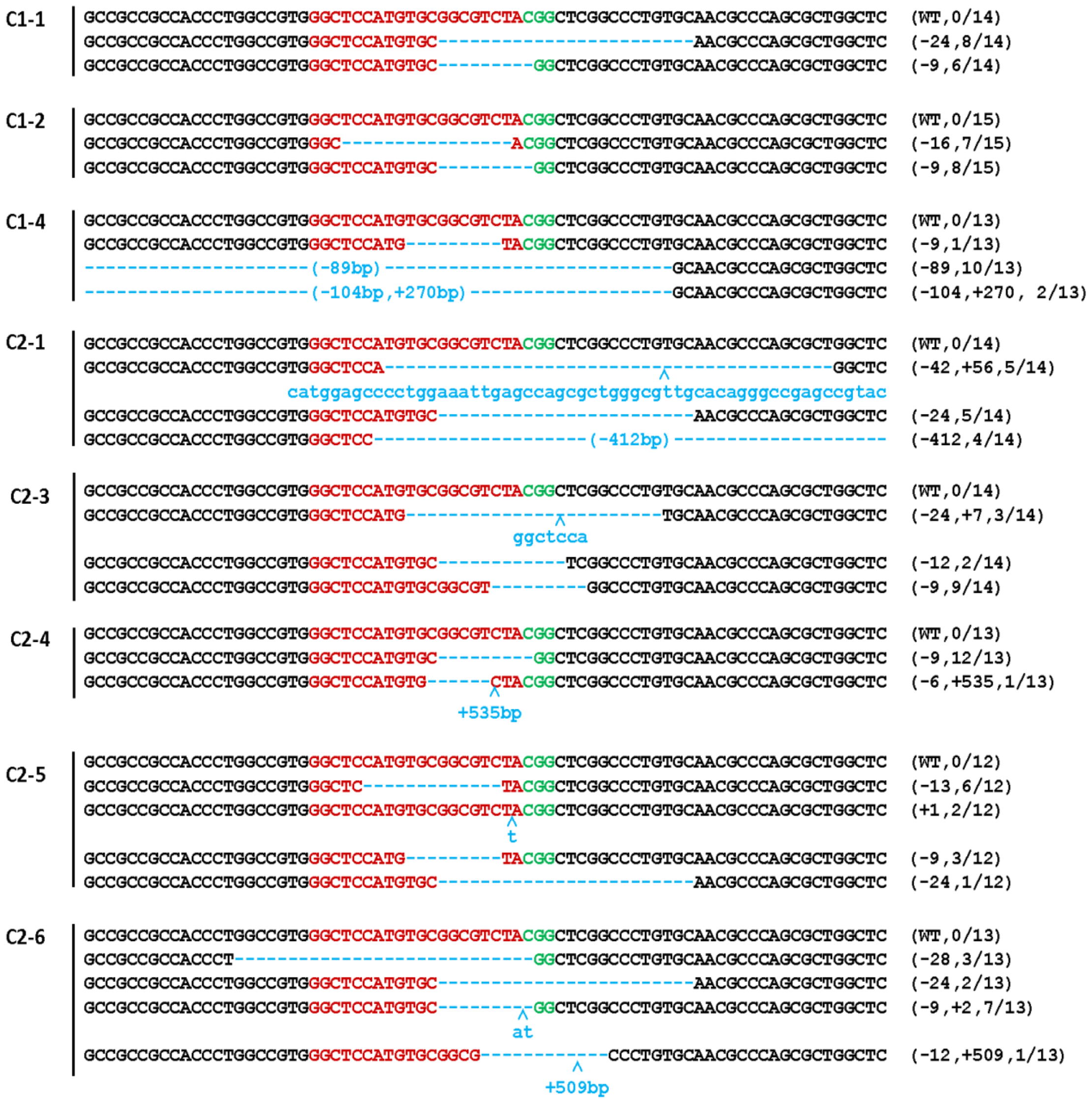

Figure S1 Sequences of the modified *Npc1l1* alleles detected in the live founder pigs

Sequencing results of the modified *Npc1l1* alleles detected in the live founder pigs. PCR products of the targeted region of *Npc1l1* were amplified from all the founder pigs and sequenced. The PAM sequences are highlighted in green; the targeting sequences in red; the mutations in blue, lower case; deletions (-), and insertions (+). N/N indicates positive colonies out of total sequenced.

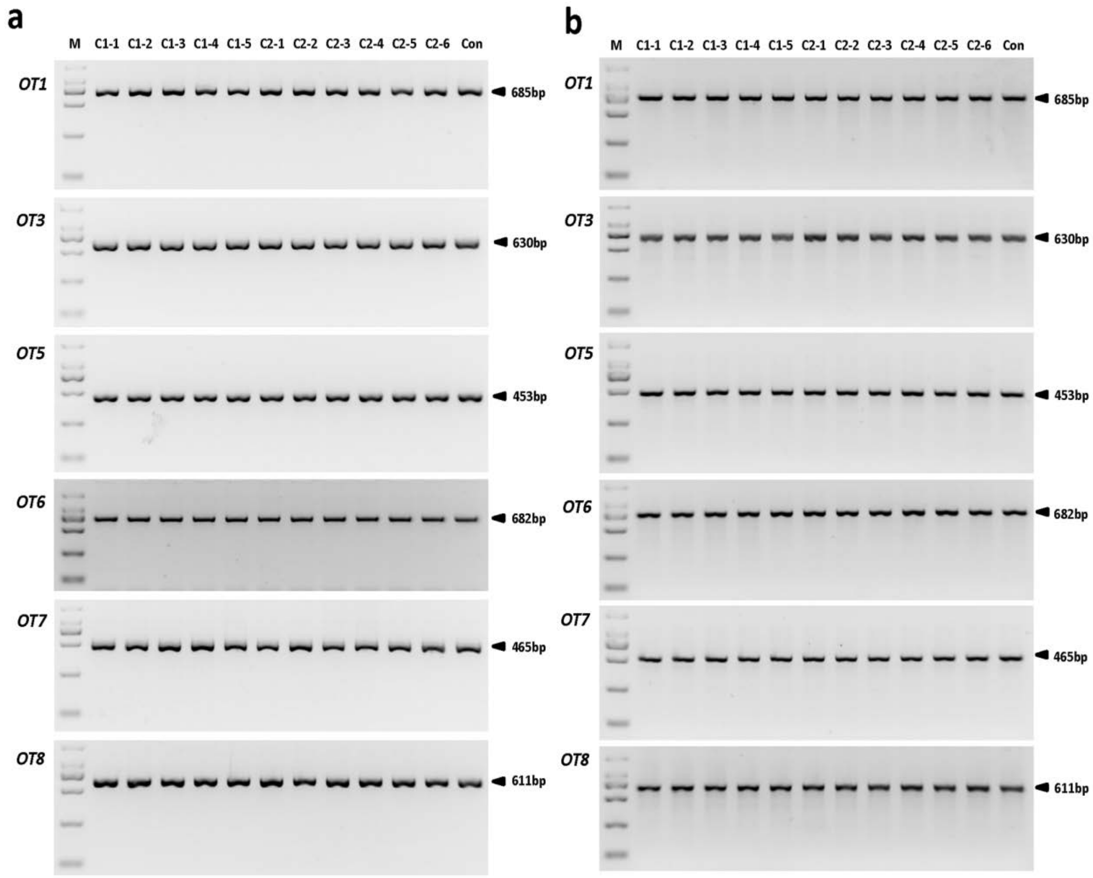

Figure S2 Detection of the *Npc1l1* sgRNA:Cas9-mediated off-target cleavages *in vivo*

(a) PCR products of the potential off-target sites of *Npc1l1* sgRNA:Cas9 from founder pigs. A total of 8 potential off-target sites most homologous to *Npc1l1* sgRNA were named OT1 to OT8. OT1, 3, 5~8 were subjected to PCR amplification using genomic DNA from founders.

(b) Detection of *Npc1l1* sgRNA:Cas9-mediated off-target cleavage by T7EN1 cleavage assay. All PCR products from (a) were subjected to T7EN1 cleavage assay.

Supplementary Table 1 List of putative off-target sites homologous to *Npc1l1*-sgRNA

Position	20	19	18	17	16	15	14	13	12	11	10	9	8	7	6	5	4	3	2	1	N	G	G	Location			
pNpc1L1	G	G	C	T	C	C	A	T	G	T	G	C	G	G	C	G	T	C	T	A	C	G	G	Chro.	Strand	Start	End
OT1	G	G	C	T	C	C	A	T	G	T	G	C	A	G	A	G	G	C	T	A	C	A	G	1	+	55561919	55561942
OT2	G	G	C	T	C	T	G	T	T	T	G	C	G	G	C	G	T	C	T	A	A	A	G	6	-	151976910	151976933
OT3	G	G	C	T	G	C	A	T	G	T	G	C	G	G	C	G	T	A	T	G	G	A	G	7	+	53582684	53582707
OT4	G	G	C	C	C	C	G	T	G	C	G	C	A	G	C	G	T	C	T	A	C	A	G	8	-	631994	632017
OT5	G	G	C	T	C	C	A	C	C	T	G	C	G	G	C	G	T	A	T	A	G	A	G	9	+	8212187	8212210
OT6	G	G	C	T	C	C	A	T	G	G	G	C	G	G	C	G	C	C	T	C	C	G	G	10	+	44897687	44897710
OT7	T	G	C	T	C	C	A	T	G	C	C	C	T	G	C	G	T	C	T	A	C	A	G	16	-	82099593	82099616
OT8	G	G	C	T	A	C	A	T	G	T	G	C	T	G	C	G	T	C	T	T	C	A	G	18	-	23387983	23388006

PAM is shaded in pink, NAG is highlighted. Bases identical to sgRNA are shaded in red.

Supplementary Table 2 Oligonucleotides for generating *Npc1l1*-sgRNA expression vector

Npc1l1-sgRNA top strand	5’-TAGGCTCCATGTGCGGCGTCTA
Npc1l1-sgRNA bottom strand	5’-AAACTAGACGCCGCACATGGAG

Supplementary Table 3 Primers for genotyping and amplifying Cas9/sgRNA-targeted *Npc1l1* fragment

Name	Sequence	Amplicon
pNpc1l1 For	5’-TGCATTGCCACAACACCTGCAGC	648
pNpc1l1 Rev	5’-TGCTAAGGAGGCTGTAGGTGGAG	

Supplementary Table 4 Sequences of primers for PCR amplification of *Npc1l1* sgRNA:Cas9 off-target sites

Name	Sequence	Amplicon
OT1 For	5′-GGAGACACCTTGAGACTTCAGA	685
OT1 Rev	5′-CCAGGAACTTGTGAGGAATGC	
OT2 For	5′-ATCTGGTCGTCTGTCTTAGGTT	681
OT2 Rev	5′-GCACAGGTATTCTCATCACAATC	
OT3 For	5′-AGACTGCCAGCAGACAACCAA	630
OT3 Rev	5′-GAGCCGCATCTGTGACCTACA	
OT4 For	5′-AGGCAACAGTTGGCATCCATAAC	757
OT4 Rev	5′-TGAGATGAAGACGGAGCGACTC	
OT5 For	5′-CTATGAAGACAGTGAATGACTC	453
OT5 Rev	5′-CAGGATAGAAGTTCCCGACAT	
OT6 For	5′-TTGAGTGAGTGTGAATGTTCTG	682
OT6 Rev	5′-CAGTCCAAAGCATAACCTAATC	
OT7 For	5′-GCTTCCTTTCCGACTGCCTATGT	465
OT7 Rev	5′-GGTGTGCTGCTCGTGCCTAAT	
OT8 For	5′-TGGAGACCTGACTTCATCTAGG	611
OT8 Rev	5′-GGCATCTGAGATAGGAGCATTC	
